# Craniofacial and Craniocervical Features in Cartilage-Hair Hypoplasia: A Radiological Study of 17 Patients and 34 Controls

**DOI:** 10.3389/fendo.2021.741548

**Published:** 2021-12-10

**Authors:** Heidi Arponen, Marjut Evälahti, Outi Mäkitie

**Affiliations:** ^1^ Department of Oral and Maxillofacial Diseases, University of Helsinki, Helsinki, Finland; ^2^ Children’s Hospital, Pediatric Research Center, University of Helsinki and Helsinki University Hospital, Helsinki, Finland; ^3^ Research Program for Clinical and Molecular Metabolism, Faculty of Medicine, University of Helsinki, Helsinki, Finland; ^4^ Folkhälsan Research Center, Helsinki, Finland; ^5^ Department of Molecular Medicine and Surgery, Karolinska Institutet and Clinical Genetics, Karolinska University Hospital, Stockholm, Sweden

**Keywords:** Cartilage-hair hypoplasia, chondrodysplasia, craniofacial, basilar invagination, cephalometrics

## Abstract

**Background:**

Biallelic mutations in the non-coding RNA gene *RMRP* cause Cartilage-hair hypoplasia (CHH), a rare skeletal dysplasia in which the main phenotypic characteristic is severe progressive growth retardation.

**Objective:**

This study compared the cranial dimensions of individuals with CHH to healthy subjects.

**Methods:**

Lateral skull radiographs of 17 patients with CHH (age range 10 to 59 years) and 34 healthy individuals (age range 10 to 54 years) were analyzed for relative position of the jaws to skull base, craniofacial height and depth, as well as vertical growth pattern of the lower jaw, anterior cranial base angle, and the relationship between the cervical spine and skull base.

**Results:**

We found that the length of the upper and lower jaws, and clivus were significantly decreased in patients with CHH as compared to the controls. Anterior cranial base angle was large in patients with CHH. Basilar invagination was not found.

**Conclusion:**

This study found no severe craniofacial involvement of patients with CHH, except for the short jaws. Unexpectedly, mandibular deficiency did not lead to skeletal class II malocclusion.

**Clinical Impact:**

Although the jaws were shorter in patients with CHH, they were proportional to each other. A short posterior cranial base was not associated with craniocervical junction pathology.

## Highlights

**Key Finding:** Cartilage-hair hypoplasia (CHH) is a rare inherited chondrodysplasia characterized by severe growth disturbance. This study found a reduced lower facial depth in individuals with CHH as compared to healthy controls. Craniocervical junction pathology was not detected.

**Importance:** This study is the largest presentation to date on craniofacial characteristics of adult individuals with Cartilage-hair hypoplasia.

## Introduction

Cartilage-hair hypoplasia (CHH) is a rare autosomal recessive chondrodysplasia caused by mutations in the untranslated *RMRP* gene on chromosome 9p13-p12 encoding the RNA component of a mitochondrial ribonuclease complex (1). It affects bone metaphyses and consequently leads to progressive growth disturbance and severe short-limbed short stature (2). The adult heights range from 110-140 cm (3). Vertebral bodies show only mild abnormalities, thereby leading to a characteristic disproportion of a long trunk in relation to the short limbs (4). Lumbar lordosis and mild scoliosis are present in some individuals (4). Patients have thin hair, variable immune deficiency and joint laxity (2, 5). CHH is exceptionally prevalent in Finland and among the North American Amish-population (2, 6). The incidence in Finland is estimated to be 1:23 000 live births (4).

To date, only one report has been published on the radiologically evaluated craniofacial morphology of 19 CHH patients aged between 8 to 22 years (7). Findings of studies on a rare disorder require several investigations to verify the results and determine how they can be generalized to the whole patient population. The aim of this cross-sectional radiological study was to examine the craniofacial and craniocervical progression characteristics of individuals with CHH.

## Methods

### Editorial Policies and Ethical Considerations

This study was conducted according to the ethical regulations of the University of Helsinki and Helsinki University Hospital (ethical approval ID 836/2018), and conforms to the STROBE guidelines. Informed consent was obtained from all the participants with CHH.

Control images were randomly collected from radiographic records of participants and participants’ parents of the Helsinki Longitudinal Growth Study (8). This prospective growth study was conducted between 1967 and 1994, and approved by the Ethics Committee of the Institute of Dentistry, University of Helsinki. Age matched controls, within a three-month range, were selected for the one patient with a radiograph obtained during growth.

### Material and Methods

We identified altogether 97 adult CHH patients currently or previously followed at Helsinki University Hospital who were eligible for the study. Inclusion criterion was a confirmed diagnosis of CHH. Growing patients under the age of 18 were excluded from the study to limit exposure to ionizing radiation. In total, 17 eligible individuals were able to participate in this study which coincided with the global SARS-CoV-2 pandemic outbreak hindering the participation of other willing candidates (**Table 1**). For the study, a single lateral skull radiograph was obtained of 16 participants. In addition, one previously taken radiograph from childhood of an additional participant was included in the study.

**Table 1:** Cephalometric measurement average (and standard deviation) for patients and healthy controls.

	Patients N = 17	Controls N = 34
Age average (range)	40.4 (9.8-59.0)	38.4 (9.5-53.9)
SNA angle	80.4 (4.2)	82.2 (3.3)
SNB angle	77.4 (3.7)	79.4 (4.3)
ANB angle	3.1 (3.6)	2.8 (2.6)
SN/MP angle	31.5 (6.9)	30.2 (7.5)
ANS-PNS	48.7 (3.3)*	50.6 (3.0)
Co-Pgn	109.3 (6.5)**	115.7 (6.0)
Harvold difference	24.4 (6.6)	27.1 (4.7)
N-Me	112.2 (8.1)	114.4 (6.3)
S-N	69.0 (4.2)	69.1 (2.8)
S-Ba	40.8 (4.7)*	43.6 (3.3)
S-N-Ba angle	133.9 (3.7)**	129.6 (5.3)

**Figure 1:**
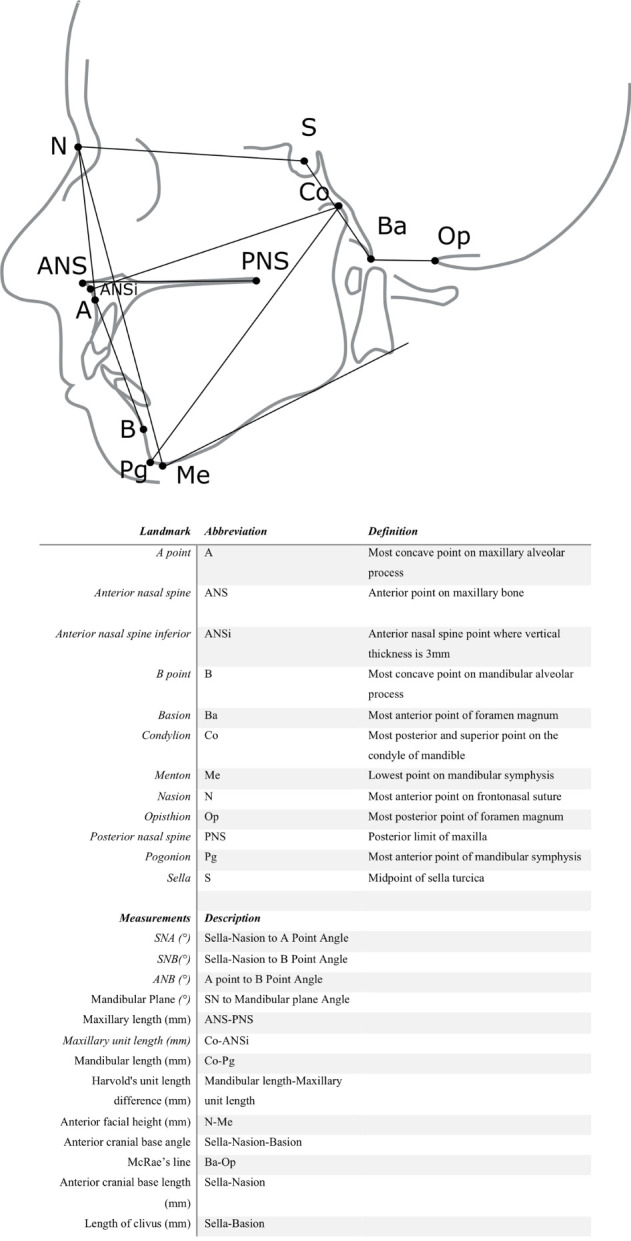
Anatomic landmarks, cephalometric reference planes, linear, and angular measurements.

Angular measurement values are presented as degrees and linear measurement values as millimeters. Measurements are explained in detail in **Figure 1**.

*Statistically significant difference, at the p< 0.05 level, between the patient and control groups, t-test.

**Statistically significant difference, at the p< 0.01 level, between the patient and control groups, t-test.

SNA, Sella-Nasion to A Point; SNB, Sella-Nasion to B Point; ANB, A point to B Point; SN/MP, Sella-Nasion plane to Mandibular plane; ANS-PNS, Anterior nasal spine to Posterior nasal spine length; Co-Pg, Condylion to Pogonion length; Harvold difference, Maxillomandibular difference (obtained reducing the length of the Condylion-Anterior nasal spine of the Condylion-Pogonion length); N-Me, Nasion to Menton length; S-N, Sella to Nasion length; S-Ba, Sella to basion length; S-N-Ba, Sella to Nasion to Basion.

Lateral skull radiographs of the individuals with CHH and healthy controls were analyzed by a single experienced examiner to assess craniofacial height and depth, along with the vertical growth pattern of the lower jaw and craniocervical junction anatomy. **Figure 1** displays the cephalometric landmarks, reference planes, and angular measurements applied.

The relationship of the maxilla and mandible to the skull base were evaluated with Steiner analysis (9). Facial height was calculated as the linear distance from the frontonasal suture to the lower border of the mandible. Length of maxilla and mandible, as measured from the most superior point on the head of the condyle to the most anterior-inferior point on the chin, was calculated and the proportion of jaw lengths was evaluated with Harvold unit length difference (10). The anterior cranial base angle was measured, and the standard McRae’s line was determined to evaluate the relationship between the occipital bone and cervical spine. McRae’s line is drawn across the foramen magnum and the tip of the odontoid process of C2 should normally be below this line (11).

The tracings were repeated in a random order after a minimum time of one week. Seven of the eleven measurements were used to test for consistency between the two time points (intra-rater reliability) using a two-way mixed-effects model. The four measurements that were not repeated (ANB angle, anterior cranial base angle, anterior cranial base length, and length of the clivus) derive from the same cephalometric landmarks as those with repeated measurements. A single examiner conducted all the analysis as intra-examiner reliability of landmark identification in cephalometric analysis has been found to be greater than inter-examiner reliability (12). Radiographic magnification was corrected for natural size in each image. Radiographic magnification was unknown for one image, of which only angular measurements were conducted.

Statistical analyses were performed with SPSS software (IBM^®^, version 23). Kolmogrov-Smirnov and Shapiro-Wilks tests of normality were carried out to test the distribution of the data. The differences between the patient and control groups were analyzed statistically with a t-test. Spearman’s rank correlation was applied to test the association between the measurements in the patient group.

## Results

Radiographs of 17 patients (5 males, 12 females) diagnosed with CHH were included in the study. One of the radiographs was of a growing individual (9.8 years). The mean age of the other patients was 42.4 years (range 26.5 to 59 years). The control group consisted of 34 healthy individuals (10 males, 24 females) with a mean age of 38.4 years (range 9.5 to 53.9 years). Two of the control images were of growing individuals age- and gender-matched with the child with CHH.

### Method Reliability

A high degree of reliability was found between SNA, SNB, Sella-Nasion to mandibular plane angle (SN/MP), face height, and jaw length measurements. The average measure intra-class correlation coefficient (ICC) for the SNA angle measurements was 0.877, with a 95% confidence interval ranging from 0.785 to 0.930 (F(50,50)=8.150, p<0.001). The average ICC for the SNB angle measurements was 0.964 (CI 0.937–0.980), (F(50,50)=27.949, p<0.001). The average ICC for the SN/MP angle was 0.813 (CI 0.672–0.893), (F(50,50)=5.336, p<0.001). The average ICC for the anterior facial height measurement was ICC 0.985(CI 0.974–0.992), (F(49,49)=4.081, p<0.001). The average ICC for the maxillary length measurement was ICC 0.755(CI 0.568–0.861), (F(49,49)=4.081, p<0.001). The average ICC for the mandibular length measurement was ICC 0.932(CI 0.881–0.962), (F(49,49)=14.807, p<0.001). Moderate reliability was found for the Harvold unit length difference measurements, with an ICC of 0.744 (CI 0.549–0.855), (F(49,49)=3.903, p<0.001).

### Craniofacial Dimensions

**Figure 2:**
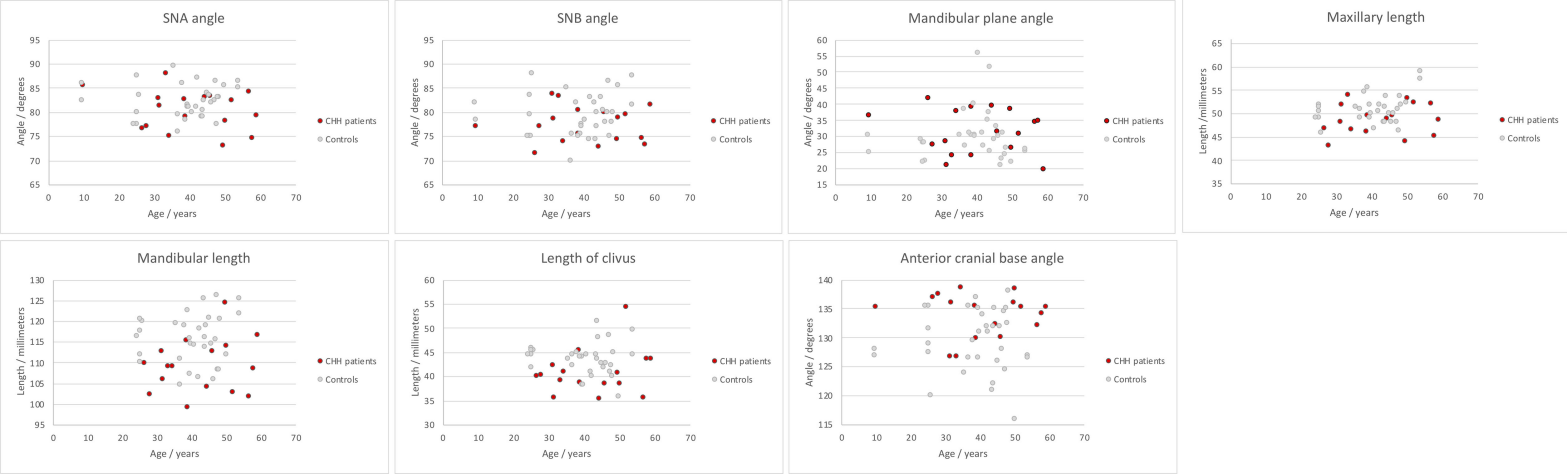
Distribution of cephalometric measurements in patients with Cartilage-hair hypoplasia (CHH) and heathy controls.

Radiographic evaluation revealed that the maxilla and mandible of adults were shorter in 65% and 76% of the patients with CHH, respectively, as compared to the controls (**Figure 2**). In one patient, the length of the maxilla was below 2SD from the mean of the controls. The mean difference in maxillary length (1.8mm) and mandibular length (6.5mm) was statistically significant (p=0.03 and p=0.001 respectively). In one patient, the mandibular length was below 2SD from the mean of the controls. The unit length difference between the jaws was smaller in patients with CHH as compared to the controls, reflecting the short mandible, but the difference was statistically insignificant. As expected, mandibular length was correlated to the retrusive position of the mandible relative to skull base (r_s_=0.470, p=0.001).

Anterior facial height was shorter in the patient group by an average of 2.1mm, however the difference between the patient and control groups was statistically insignificant. Similarly, there were no statistically significant differences in the angular measurements between the patients and controls (**Table 1**).

### Cranial Base Dimensions

Length of the anterior cranial base was similar in individuals with CHH and controls (p>0.72). The length of the clivus was significantly shorter in the patient group as compared to the controls by an average of 2.8mm (p=0.02). Length of the clivus was correlated with length of the lower jaw (r_s_=0.482, p<0.001) and anterior facial height (r_s_=0.449, p=0.001).

The anterior cranial base angle was significantly larger in patients with CHH by an average of 4 degrees compared to the controls (p=0.004). None of the patients displayed an anterior cranial base angle measurement that exceeded the previously documented abnormality threshold value (143 degrees) for the same ethnicity (13). None of the patients or controls exhibited an abnormally high position of the odontoid process above the foramen magnum level, i.e. basilar invagination, as evaluated by the McRae measure.

## Discussion

This study makes an original contribution to the knowledge on characteristic craniofacial and cranial base features of patients with CHH. Our study found that the upper and lower jaw of patients with CHH are shorter, and the anterior cranial base is more obtuse than in healthy individuals. The length of the clivus at the skull base was shorter and associated with a short facial height in patients with CHH as compared to healthy controls. Length of the lower jaw was more significantly affected than that of the upper jaw in patients with CHH. However, the relative position of the upper and lower jaws to the skull base were unaffected in patients with CHH, as was the vertical growth pattern of the lower jaw.

Cranial base synchondroses are growth centers of the craniofacial skeleton, composed of primary cartilage, and are the last sites in the cranium to complete growth (7). Among the four cranial base synchondroses, the spheno-occipital synchondrosis is the most important site for sagittal and vertical growth that continues until puberty (14). Growth at the sphenoethmoidale and frontoethmoid synchondroses is completed by the age of seven years (15). During normal growth, the anterior cranial base angle steepens moderately, and the jaws are displaced downward and forward (16, 17). Elongation of the cranial base moves the glenoid fossa on the temporal bone inferiorly and posteriorly, which holds important implications for mandibular displacement (18). An obtuse cranial base angle shifts the mandible in a backward retrognathic position relative to the maxilla (16). The maxilla mainly grows by bone apposition at the sutures that connect the maxilla to the cranium and the cranial base, whereas mandibular growth relies on appositional proliferation at condylar secondary cartilage (19).

Disturbance of cartilage growth in CHH is selective (2, 3, 7). The mean relative head circumference of patients with CHH is normal or slightly smaller than that of healthy controls (3). Cranial base cartilage has been shown to be affected by this disorder, although the findings have been inconsistent (2, 7, 20). In the sole previously published study that features patients with CHH, the depth dimension of the middle face was similar to that of controls, whereas the lower face was found to be small, with a short sella-basion distance and large angle between the sphenoidal bone and clivus (7). Our study verified the report of an obtuse cranial base. In light of the unusually large cranial base angle, our finding of an inconspicuous deviation in the relationship between the jaws of patients with CHH from normal was unexpected.

Deviation from normal mandible growth can affect masticatory functions, speech, and make the affected individual susceptible to obstructive sleep apnea (21, 22). Rönning and co-workers described the feature of a receding chin in patients with CHH. The reduced mandibular length found in this study is clinically significant and might be a consequence of growth failure at the mandibular condyle. Interestingly, however, we found that in patients with CHH, the position of the lower jaw was normal in relation to the upper jaw and skull base. This finding could be explained by a more anterior position of the temporomandibular articulation due to a decreased cranial base length – a theory supported by the findings of Rönning and co-workers, who described a short clivus and an unusually low position of the mandibular condyle in patients with CHH (7). Our findings are in agreement with those of Rönning, and reflect subnormal growth at the spheno-occipital synchondrosis resulting in a short clivus in patients with CHH (7). Previous studies have shown that function of the masticatory system is impaired in healthy retrognathic individuals with a class II malocclusion (22). While masticatory function of individuals with CHH has not been examined in previous or present study, our finding of a normal position of the lower jaw in relation to the upper jaw and skull base eliminates a skeletal predisposing factor for functional impairment.

Our findings of normal facial height in patients with CHH are contradictory to those of Rönning and colleagues, who reported a large facial height in patients with CHH. This inconsistency might be explained by the fact that our study examined facial height in adults only in whom the progressive growth failure is expected to be more evident than in children.

Studies on other chondrodysplasias, such as achondroplasia and diastrophic dysplasia, have revealed several similar facial features (23–25). Midfacial hypoplasia leads to the characteristic dysmorphology of achondroplasia (25). Whereas, for example, in diastrophic dysplasia and spondyloepiphyseal dysplasia the lower jaw is typically more pronouncedly affected and unusually short (24). This study indicates a small lower jaw as a craniofacial feature of CHH. Basilar pathology is also often encountered in genetic disorders of the skeleton, and a short clivus has been suggested to play a role in the pathophysiology of basilar invagination (26, 27). Contradictory results on cervical spine instability and deformity have been described in patients with CHH (28, 29). Our study found no craniocervical junction pathology in the patient group despite the short clivus.

The strength of this study is that the observations were carried out by a single investigator on an ethnically homogenous population. Radiographs of the patients were digitally traced and analyzed, whereas those of controls were manually traced. Both digital and manual tracing methods have been shown to provide similar clinical results (30). Although we repeated the tracings of all the radiographs, we cannot rule out the possibility of a systematic examiner-dependent error or an error caused by larger variability in the quality of the conventional film radiographs in some patients.

The findings of this study are in agreement with those of a previous one examining craniofacial dimensions of growing individuals with CHH and therefore imply generalizability of our findings to the whole CHH population. Craniofacial anthropometry has been recommended as a potentially useful tool in differential diagnostics of chondrodysplasias (24). Our study indicates reduced lower facial depth as a feature of CHH.

## Data Availability Statement

The original contributions presented in the study are included in the article/supplementary material. Further inquiries can be directed to the corresponding author.

## Ethics Statement

The studies involving human participants were reviewed and approved by Helsinki University Hospital (ethical approval ID HUS836/2018). Written informed consent to participate in this study was provided by the participants or (where required) the participants’ legal guardian/next of kin.

## Author Contributions

Study design: HA and OM. Acquisition of data: HA and ME. Data analysis: HA. Interpretation of data: HA and OM. Drafting manuscript: HA. Revising manuscript content: HA, ME, and OM. All authors agree to be accountable for the content of the work. All authors contributed to the article and approved the submitted version.

## Funding

This work was supported by: Academy of Finland (grant number: 332585) and Finnish Dental Society (personal grant to HA). Foundation for Pediatric Research, Helsinki University Hospital Research Funds, and Sigrid Jusélius Foundation (grant to OM). Helsinki University Library (funds for open access publication fees).

## Conflict of Interest

The authors declare that the research was conducted in the absence of any commercial or financial relationships that could be construed as a potential conflict of interest.

## Publisher’s Note

All claims expressed in this article are solely those of the authors and do not necessarily represent those of their affiliated organizations, or those of the publisher, the editors and the reviewers. Any product that may be evaluated in this article, or claim that may be made by its manufacturer, is not guaranteed or endorsed by the publisher.
